# Determination of HLA‐A, ‐C, ‐B, ‐DRB1 allele and haplotype frequency in Japanese population based on family study

**DOI:** 10.1111/tan.12536

**Published:** 2015-02-27

**Authors:** N. Ikeda, H. Kojima, M. Nishikawa, K. Hayashi, T. Futagami, T. Tsujino, Y. Kusunoki, N. Fujii, S. Suegami, Y. Miyazaki, D. Middleton, H. Tanaka, H. Saji

**Affiliations:** ^1^ HLA Foundation LaboratoryKyotoJapan; ^2^ Transplant ImmunologyRoyal Liverpool University Hospital Trust and University of LiverpoolLiverpoolUK

**Keywords:** allele frequency, family study, haplotype frequency, human leucocyte antigen, Japanese population

## Abstract

The present study investigates the human leucocyte antigen (HLA) allele and haplotype frequencies in Japanese population. We carried out the frequency analysis in 5824 families living across Japanese archipelago. The studied population has mainly been typed for the purpose of transplant, especially the hematopoietic stem cell transplantation (HSCT). We determined HLA class I (A, B, and C) and HLA class II (DRB1) using Luminex technology. The haplotypes were directly counted by segregation. A total of 44 HLA‐A, 29 HLA‐C, 75 HLA‐B, and 42 HLA‐DRB1 alleles were identified. In the HLA haplotypes of A‐C‐B‐DRB1 and C‐B, the pattern of linkage disequilibrium peculiar to Japanese population has been confirmed. Moreover, the haplotype frequencies based on family study was compared with the frequencies estimated by maximum likelihood estimation (MLE), and the equivalent results were obtained. The allele and haplotype frequencies obtained in this study could be useful for anthropology, transplantation therapy, and disease association studies.

## Introduction

The human leukocyte antigen (HLA) gene family is characterized by extreme degree of genetic polymorphism and linkage disequilibrium (LD). The varieties in polymorphism and LD patterns of HLA gene family show a tendency to be unique in each ethnic group 1, 2. HLA antigens have been known to play an important role in immune responses. In hematopoietic stem cell transplantation (HSCT), HLA matching between donors and recipients lowers the risk of graft rejection and graft‐versus‐host disease (GVHD) 3, 4. Morishima et al. suggested that the genetic difference derived from HLA haplotype is associated with acute GVHD in allogeneic HSCT 5. Therefore, HLA haplotype cannot be excluded from consideration during donor selection because of potential contribution from proteins encoded by non‐HLA genes inherited with HLA genes.

Several studies reported analysis of HLA allele and haplotype frequency data in the Japanese population 6, 7, 8, 9. However, these studies failed to present accurate and detailed information due to the haplotypes estimation using software or small sample size, or both. This necessitates developing a method which can produce accurate and detailed gene distribution. The present study aims to obtain a more exact and detailed HLA haplotype distribution from 18,604 members of 5824 Japanese families, whose HLA haplotypes were determined by descent. Our study also attempts to determine the frequency of specific haplotypes, C‐B, A‐B‐DRB1, and A‐C‐B‐DRB1, used in donor search. In addition, it was ascertained whether the haplotype frequencies estimated by maximum likelihood estimation (MLE) would be equivalent to the frequencies found in the present family study.

## Materials and method

### Subjects

A total of 18,604 members (including patients and normal subjects) from 5824 families, distributed in all parts of Japan, were enrolled for this study. Among these families, there were patients, considered for transplantation, especially HSCT. The families were divided into three groups (Table 1): (i) families with both parents with one or more children, (ii) families with one parent with one or more children, and (iii) families with no parents but having two or more children. The families with more than two generations were counted as separate families. Informed consent was obtained from all the participants of this study by the clinicians who ordered HLA typing.

**Table 1:** Breakdown of the family structure

	Number of children	Number of families^a^
Parents	1–5	2077
One parent	1–8	1968
No parent	2–7	1779
	Total	5824

a The families with more than two generations are counted as separate families.

For comparing the haplotype frequencies obtained by family study and using mle software, unrelated 4500 people were chosen at random from the total subjects of present study (18,604 members). They were genetically unrelated, because one person was chosen from each of 4500 random chosen families. The overlaps of blood relationship with three or more generation were avoided in these families. The allele and haplotype frequencies calculated from these 4500 people were very similar to the frequencies from the total subjects [allele frequencies (AF) data not shown].

### Samples

DNA samples were obtained from peripheral lymphocytes or buccal cells using a JetQuick^®^ Blood & Cell Culture Kits (GENOMED, Löhne, Germany) or QuickGene DNA Tissue Kit (KURABO, Osaka, Japan) according to the manufacturer's protocols.

### HLA allele typing

HLA (‐A, ‐C, ‐B, and ‐DRB1) four‐digit allele typing was performed using Luminex 200 system (Luminex, Austin, TX) and WAKFlow HLA Typing kit (Wakunaga, Hiroshima, Japan) 10, 11, 12. HLA alleles were assigned automatically using wakflow Typing software (Wakunaga, Hiroshima, Japan). The primer sequences of wakflow Typing kit are specifically designed to make allele determination easier in Japanese population, and by default the analysis with wakflow Typing software is based on the AF of the donors registered with Japan Marrow Donor Program (JMDP) which are available on the website, www.bmdc.jrc.or.jp 9. Therefore, this method can determine alleles with frequencies of 0.1% and greater in the Japanese population. A few alleles which could not be determined by this method as rare alleles were considered as secondary; these alleles were determined using Luminex 200 system and LAB Type SSO kit (One Lambda, Los Angeles, CA) assigned using the HLA Fusion software (One Lambda). In brief, exon 2 for HLA‐DRB1; exons 2 and 3 for HLA‐A, ‐B, and ‐C were amplified in these methods.

### Haplotype determination

The haplotypes were determined by segregation. This study was designed with the aim to assess genetic linkage with high certainty. Nevertheless, the results also included some partial haplotypes because of the possibility of one or more recombination in a family. The haplotype frequencies were calculated by using haplotypes without taking into account the recombination. Thus, we counted the haplotypes of parents and not of children because fathers and mothers are genetically unrelated, while the haplotypes created by recombination were those of children only. Some haplotypes of parents whose children had recombinant haplotypes could not been determined because of two patterns of their combinations. These haplotypes were determined and counted as not less frequent but frequent haplotype phase. Specifically, assuming that four haplotypes are Hp1, Hp2, Hp3, and Hp4, those frequencies are HF1, HF2, HF3, and HF4, and two estimable phases are ‘Hp1, Hp2’ and ‘Hp3, Hp4’, HF1 was multiplied by HF2, HF3 was multiplied by HF4, and the haplotypes of the estimable phase with larger product were counted.

For the comparison to the result by MLE, the haplo.em program, which was evaluated by haplo.stats (version 1.6.0) software operated in the R language, was used 13, 14, 15, 16. For genetic markers measured on unrelated subjects with linkage phase unknown, this program computes the maximum likelihood estimates of haplotype probabilities using the progressive insertion algorithm that progressively inserts batches of loci into haplotypes of growing lengths.

### Statistical analysis

**Figure 1:**
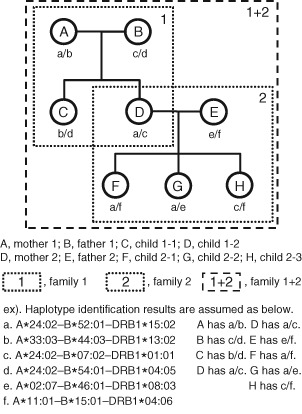
Avoiding haplotype duplication. The extended haplotypes of a and c are redundant in families 1 and 2. In this case, six haplotypes were counted as family 1 + 2.

The haplotypes were counted manually using Microsoft Excel^®^ spreadsheets. The haplotypes that extended three or more generations were counted once (Figure 1). The allele and haplotype frequencies were calculated by using 19,183 haplotypes, counted as mentioned above. Relative LD values (RD) were computed for each haplotypes 17, 18. The exact test for deviation from Hardy–Weinberg Equilibrium were evaluated by genepop software, version 4.2 19, 20, which uses a Markov Chain (MC) algorithm (dememorization = 10,000, batches = 10,000, and iterations per batch = 10,000) to estimate the *P*‐value.

The expected prevalence (P) of the allele or the haplotype under Hardy–Weinberg proportions were calculated from AF by using the following equation: P = 1 − (1 − AF)^2^.

## Results

### HLA‐A, ‐B, ‐C, and ‐DRB1 AF

Table 2 presents the list of the AF of HLA‐A, ‐B, ‐C, and ‐DRB1 loci 21. We identified 44 HLA‐A, 75 HLA‐B, 29 HLA‐C, and 42 HLA‐DRB1 alleles and found A*24:02 to be 36.48%, the highest in Japanese population; thus, it is distributed in approximately 60% of the population. The alleles underlined in Table 2 need specific attention for HLA allele matching in unrelated HSCT between the Japanese, as they are present at high frequencies within a serotype (allele family) match.

**Table 2:** HLA‐A, ‐C, ‐B, ‐DRB1 allele frequencies in Japan^a^

HLA‐A serological specificity	HLA‐A allele	GF (%)	HLA‐C serological specificity	HLA‐C allele	GF (%)	HLA‐B serological specificity	HLA‐B allele	GF (%)	HLA‐DRB1 serological specificity	HLADRB1 allele	GF (%)
A1	A*01:01	0.401	Cw1	C*01:02	17.604	B7	B*07:02	5.630	DR1	DRB1*01:01	5.823
A2	A*02:01	11.620		C*01:03	0.318		B*07:05	0.026		DRB1*01:02	0.016
	A*02:06	9.081	Cw2	C*02:02	0.026		B*07:31	0.005	DR4	DRB1*04:05	13.491
	A*02:07	3.461	Cw4	C*04:01	4.410	B8	B*08:01	0.016		DRB1*04:06	3.388
	A*02:18	0.078	Cw5	C*05:01	0.381	B13	B*13:01	1.173		DRB1*04:03	3.055
	A*02:28	0.010	Cw6	C*06:02	0.808		B*13:02	0.287		DRB1*04:10	2.153
	A*02:05	0.005	Cw7	C*07:02	12.714	B18	B*18:01	0.005		DRB1*04:01	0.954
	A*02:13	0.005		C*07:04	0.928	B22	B*56:03	0.177		DRB1*04:07	0.573
A203	A*02:03	0.063		C*07:01	0.073		B*55:12	0.005		DRB1*04:04	0.255
A210	A*02:10	0.386	Cw8	C*08:01	7.355	B27	B*27:04	0.188		DRB1*04:02	0.005
A3	A*03:01	0.396		C*08:03	1.361		B*27:05	0.063		DRB1*04:11	0.005
	A*03:02	0.094		C*08:02	0.026		B*27:06	0.005	DR6	DRB1*14:45	0.005
A11	A*11:01	9.117	Cw9	C*03:03	13.053	B35	B*35:01	8.263	DR7	DRB1*07:01	0.339
	A*11:02	0.219	Cw10	C*03:04	12.391		B*35:05	0.016	DR8	DRB1*08:03	8.320
	A*11:05	0.005		C*03:02	0.568		B*35:04	0.005		DRB1*08:02	4.207
A24	A*24:02	36.475	Cw12	C*12:02	11.182		B*35:35	0.005		DRB1*08:09	0.042
	A*24:20	0.709		C*12:03	0.083	B37	B*37:01	0.495	DR9	DRB1*09:01	14.283
	A*24:08	0.042	Cw14	C*14:02	6.871	B38	B*38:02	0.281	DR10	DRB1*10:01	0.474
	A*24:07	0.021		C*14:03	6.688		B*38:01	0.016	DR11	DRB1*11:01	2.518
	A*24:04	0.016	Cw15	C*15:02	3.081	B39	B*39:04	0.209		DRB1*11:06	0.016
	A*24:25	0.010		C*15:05	0.016		B*39:23	0.031		DRB1*11:08	0.005
	A*24:05	0.005		C*15:10	0.005		B*39:05	0.005	DR12	DRB1*12:01	3.774
A2403	A*24:03	0.005	Cw17	C*17:01	0.010	B3901	B*39:01	3.321		DRB1*12:02	1.814
A26	A*26:01	7.350	undefined	C*03:23	0.010	B3902	B*39:02	0.308	DR13	DRB1*13:02	5.948
	A*26:03	2.288		C*03:43	0.010	B41	B*41:02	0.005		DRB1*13:01	0.589
	A*26:02	1.809		C*07:15	0.010	B44	B*44:03	6.751		DRB1*13:07	0.005
	A*26:05	0.068		C*03:64	0.005		B*44:02	0.375	DR14	DRB1*14:54	3.274
	A*26:06	0.010		C*04:15	0.005	B45	B*45:01	0.005		DRB1*14:05	2.090
A29	A*29:01	0.010		C*16:04	0.005	B46	B*46:01	4.765		DRB1*14:06	1.303
A30	A*30:01	0.209					B*46:02	0.005		DRB1*14:07	0.109
	A*30:04	0.021				B48	B*48:01	2.878		DRB1*14:02	0.057
A31	A*31:01	8.403				B50	B*50:01	0.016		DRB1*14:12	0.026
A32	A*32:01	0.021				B51	B*51:01	8.925		DRB1*14:29	0.016
A33	A*33:03	7.507				B5102	B*51:02	0.235	DR1403	DRB1*14:03	1.569
	A*33:01	0.010				B5103	B*51:03	0.005	DR1404	DRB1*14:04	0.010
A34	A*34:01	0.010				B52	B*52:01	11.093	DR15	DRB1*15:02	10.650
A66	A*66:01	0.005				B53	B*53:01	0.005		DRB1*15:01	7.778
A68	A*68:01	0.010				B54	B*54:01	7.491		DRB1*15:04	0.005
Null	A*02:53N	0.010				B55	B*55:02	2.648	DR16	DRB1*16:02	0.897
Undefined	A*24:46	0.010					B*55:04	0.130	DR17	DRB1*03:01	0.146
	A*11:43	0.005				B56	B*56:01	0.850	undefined	DRB1*04:57	0.005
	A*24:88	0.005				B57	B*57:01	0.005		DRB1*08:23	0.005
	A*26:18	0.005				B58	B*58:01	0.579			
	A*31:11	0.005				B59	B*59:01	2.043			
						B60	B*40:01	5.348			
							B*40:07	0.010			
							B*40:52	0.005			
						B61	B*40:02	7.945			
							B*40:06	4.791			
							B*40:03	0.407			
							B*40:50	0.016			
							B*40:53	0.005			
							B*40:11	0.005			
						B62	B*15:01	7.585			
							B*15:07	0.652			
							B*15:27	0.109			
							B*15:28	0.016			
							B*15:35	0.016			
							B*15:25	0.010			
							B*15:38	0.005			
						B64	B*14:01	0.016			
						B65	B*14:02	0.010			
						B67	B*67:01	1.225			
						B71	B*15:18	1.522			
						B75	B*15:11	0.881			
							B*15:02	0.031			
						B77	B*15:13	0.005			
						B78	B*78:02	0.005			
						B81	B*81:01	0.005			
						Unknown^b^	B*54:21^b^	0.005			
						Null	B*15:26N	0.005			
						undefined	B*35:64	0.005			
							B*51:36	0.005			
							B*52:05	0.005			
							B*52:11	0.005			

GF, gene frequencies; HLA, human leucocyte antigen.

a GF are calculated by using 19,183 haplotypes counted manually. Nomenclature of serological specificities, refer to *Tissue Antigens* 2010: 75: 291–455 15.

b The allele is not in the paper 15.

### Haplotype segregation analysis based on family study

#### Haplotype frequencies of HLA‐A‐C‐B‐DRB1

Table 3 lists 60 haplotypes with frequencies higher than 0.2% in the population. Approximately, 38% of the entire Japanese population is expected to carry one or two of the five most common haplotypes.

**Table 3:** HLA–A–C–B–DRB1 haplotype frequencies in Japanese^a^

A–C–B–DRB1	HF (%)	RD	A–C–B–DRB1	HF (%)	RD
A*24:02–C*12:02–B*52:01–DRB1*15:02	8.377	0.79	A*02:07–C*01:02–B*46:01–DRB1*09:01	0.308	0.09
A*33:03–C*14:03–B*44:03–DRB1*13:02	4.473	0.75	A*33:03–C*03:02–B*58:01–DRB1*13:02	0.308	0.54
A*24:02–C*07:02–B*07:02–DRB1*01:01	3.722	0.66	A*26:01–C*03:03–B*35:01–DRB1*04:10	0.302	0.14
A*24:02–C*01:02–B*54:01–DRB1*04:05	2.539	0.33	A*02:01–C*03:03–B*15:11–DRB1*09:01	0.292	0.33
A*02:07–C*01:02–B*46:01–DRB1*08:03	1.866	0.54	A*24:02–C*04:01–B*15:01–DRB1*04:06	0.292	0.09
A*11:01–C*04:01–B*15:01–DRB1*04:06	1.345	0.40	A*24:02–C*14:02–B*51:01–DRB1*14:03	0.276	0.17
A*24:02–C*01:02–B*59:01–DRB1*04:05	1.058	0.51	A*24:02–C*03:04–B*40:01–DRB1*04:05	0.266	0.04
A*11:01–C*01:02–B*54:01–DRB1*04:05	1.001	0.13	A*26:02–C*03:03–B*15:01–DRB1*14:06	0.261	0.20
A*26:01–C*03:04–B*40:02–DRB1*09:01	0.745	0.10	A*24:02–C*03:04–B*40:02–DRB1*04:05	0.255	0.03
A*24:02–C*08:01–B*40:06–DRB1*09:01	0.709	0.14	A*02:01–C*07:02–B*07:02–DRB1*01:01	0.250	0.04
A*24:02–C*14:02–B*51:01–DRB1*09:01	0.652	0.09	A*02:06–C*07:02–B*07:02–DRB1*01:01	0.250	0.04
A*31:01–C*14:02–B*51:01–DRB1*08:02	0.579	0.14	A*11:01–C*01:02–B*54:01–DRB1*08:03	0.240	0.03
A*33:03–C*14:03–B*44:03–DRB1*08:03	0.547	0.08	A*11:01–C*01:02–B*55:02–DRB1*04:05	0.240	0.09
A*26:02–C*08:01–B*40:06–DRB1*09:01	0.542	0.30	A*31:01–C*14:02–B*51:01–DRB1*04:05	0.240	0.03
A*02:01–C*03:04–B*13:01–DRB1*12:02	0.532	0.45	A*02:01–C*15:02–B*51:01–DRB1*15:01	0.235	0.08
A*24:02–C*01:02–B*46:01–DRB1*08:03	0.532	0.11	A*03:01–C*05:01–B*44:02–DRB1*13:01	0.235	0.63
A*02:06–C*08:01–B*40:06–DRB1*09:01	0.464	0.10	A*11:01–C*07:02–B*67:01–DRB1*15:01	0.235	0.19
A*11:01–C*07:02–B*39:01–DRB1*08:03	0.433	0.13	A*24:02–C*14:03–B*44:03–DRB1*13:02	0.235	0.04
A*26:01–C*03:04–B*40:02–DRB1*08:02	0.427	0.10	A*01:01–C*06:02–B*37:01–DRB1*10:01	0.229	0.57
A*02:06–C*03:03–B*35:01–DRB1*15:01	0.422	0.05	A*24:02–C*03:03–B*35:01–DRB1*15:01	0.229	0.03
A*24:02–C*12:02–B*52:01–DRB1*09:01	0.391	0.03	A*24:02–C*03:04–B*40:01–DRB1*11:01	0.229	0.09
A*31:01–C*14:02–B*51:01–DRB1*14:03	0.391	0.25	A*31:01–C*14:02–B*51:01–DRB1*09:01	0.229	0.03
A*02:06–C*07:02–B*39:01–DRB1*15:01	0.386	0.12	A*26:03–C*03:03–B*15:01–DRB1*09:01	0.224	0.10
A*24:02–C*03:04–B*40:02–DRB1*09:01	0.370	0.04	A*02:06–C*01:02–B*54:01–DRB1*04:05	0.219	0.03
A*02:01–C*01:02–B*54:01–DRB1*04:05	0.360	0.05	A*02:06–C*03:03–B*35:01–DRB1*09:01	0.219	0.02
A*26:03–C*03:03–B*15:01–DRB1*15:01	0.344	0.15	A*02:01–C*01:02–B*46:01–DRB1*08:03	0.214	0.04
A*11:01–C*07:02–B*67:01–DRB1*16:02	0.339	0.38	A*02:01–C*08:01–B*40:06–DRB1*09:01	0.214	0.04
A*02:06–C*01:02–B*59:01–DRB1*04:05	0.323	0.16	A*02:06–C*08:01–B*48:01–DRB1*04:07	0.214	0.37
A*24:02–C*03:03–B*15:07–DRB1*04:03	0.323	0.50	A*31:01–C*04:01–B*56:01–DRB1*09:01	0.214	0.25
A*24:02–C*07:04–B*15:18–DRB1*04:01	0.318	0.34	A*31:01–C*07:02–B*07:02–DRB1*01:01	0.209	0.04

HF, haplotype frequencies; HLA, human leucocyte antigen; RD, relative linkage disequilibrium value.

a Four‐locus haplotypes with HF >0.2% are listed.

These data have also been submitted to Allele Frequency Net Database (AFND) 22. The four‐loci haplotypes with frequencies equal to or more than 0.01% and the AF can be found at the AFND website, www.allelefrequencies.net 22.

#### HLA‐A‐B‐DRB1 haplotype sets with the same serotypes

Table 4 lists the sets of three‐loci haplotypes at frequencies of 0.2% or greater, which would have the same serotype.

**Table 4:** Haplotype sets assigned same serotype of HLA–A–B–DRB1^a^

Set no.	Haplotype	HF (%)
1	A*02:01‐B*07:02‐DRB1*01:01	0.26
	A*02:06‐B*07:02‐DRB1*01:01	0.25
2	A*02:01‐B*46:01‐DRB1*08:03	0.23
	A*02:07‐B*46:01‐DRB1*08:03	1.87
3	A*02:01‐B*54:01‐DRB1*04:05	0.38
	A*02:06‐B*54:01‐DRB1*04:05	0.22
4	A*02:01‐B*40:06‐DRB1*09:01	0.29
	A*02:06‐B*40:06‐DRB1*09:01	0.48
5	A*24:02‐B*40:02‐DRB1*09:01	0.53
	A*24:02‐B*40:06‐DRB1*09:01	0.95
6	A*24:02‐B*15:01‐DRB1*04:06	0.31
	A*24:02‐B*15:07‐DRB1*04:03	0.34
7	A*24:02‐B*35:01‐DRB1*04:03	0.23
	A*24:02‐B*35:01‐DRB1*04:05	0.22
8	A*26:01‐B*40:02‐DRB1*09:01	0.81
	A*26:01‐B*40:06‐DRB1*09:01	0.22
	A*26:02‐B*40:06‐DRB1*09:01	0.54

HF, haplotype frequency; HLA, human leucocyte antigen.

a The analysis objects are HF with more than 0.2%.

#### Haplotype frequencies of HLA‐C‐B

Table 5 lists 64 haplotypes with frequencies higher than 0.1 % in the population. Half of these haplotypes have RD values more than 0.7, suggesting conservation of HLA‐C‐B linkage. B*40:02 and B*40:06 which correspond to the same serotype (B61) have high frequencies alleles of B61 and need attention for matching in HSCT. Focusing on these, Table 5 shows the frequency of C*03:04‐B*40:02 as 6.26%, while the frequency of B*40:02 is 7.95% (Table 2); therefore, the frequency of C*03:04‐B*40:02 linkage would account for 79% of B*40:02 alleles. Similarly, linkage of B*40:06 with C*08:01 would account for 81% of the presence of B*40:06. Thus, it is important to differentiate serotypes such as B61 when the constituting alleles are in linkage with different HLA‐C alleles.

**Table 5:** HLA–C–B haplotype frequencies in Japanese^a^

C–B	HF (%)	RD	C–B	HF (%)	RD
C*12:02–B*52:01	10.963	0.99	C*08:01–B*15:18	0.495	0.27
C*01:02–B*54:01	7.147	0.94	C*03:04–B*51:01	0.469	−0.58
C*14:02–B*51:01	6.761	0.98	C*04:01–B*35:01	0.464	0.02
C*14:03–B*44:03	6.641	0.99	C*04:01–B*40:01	0.464	0.05
C*03:04–B*40:02	6.256	0.76	C*03:04–B*40:03	0.396	0.97
C*03:03–B*35:01	6.115	0.70	C*05:01–B*44:02	0.370	0.99
C*07:02–B*07:02	5.573	0.99	C*03:04–B*35:01	0.339	−0.67
C*01:02–B*46:01	4.316	0.89	C*03:04–B*40:06	0.339	−0.43
C*08:01–B*40:06	3.904	0.80	C*15:02–B*40:06	0.334	0.06
C*07:02–B*39:01	3.196	0.96	C*01:03–B*46:01	0.318	1.00
C*03:03–B*15:01	3.190	0.33	C*07:02–B*39:02	0.308	1.00
C*03:04–B*40:01	2.867	0.47	C*15:02–B*40:02	0.292	0.02
C*04:01–B*15:01	2.606	0.56	C*06:02–B*13:02	0.287	1.00
C*01:02–B*55:02	2.252	0.82	C*07:02–B*38:02	0.276	0.98
C*01:02–B*59:01	2.012	0.98	C*03:04–B*15:01	0.266	−0.72
C*08:01–B*48:01	1.553	0.50	C*03:03–B*55:02	0.261	−0.25
C*15:02–B*51:01	1.345	0.38	C*03:03–B*48:01	0.240	−0.36
C*07:02–B*67:01	1.220	1.00	C*01:02–B*56:01	0.235	0.12
C*03:04–B*13:01	1.069	0.90	C*03:03–B*40:01	0.235	−0.66
C*08:01–B*35:01	1.069	0.07	C*08:03–B*54:01	0.229	0.10
C*03:03–B*40:02	1.048	0.00	C*01:02–B*51:01	0.224	−0.86
C*07:02–B*40:01	0.923	0.05	C*07:02–B*39:04	0.209	1.00
C*08:03–B*48:01	0.923	0.67	C*01:02–B*56:03	0.177	1.00
C*07:04–B*15:18	0.907	0.98	C*15:02–B*51:02	0.151	0.63
C*03:03–B*15:11	0.850	0.96	C*01:02–B*40:02	0.141	−0.90
C*15:02–B*40:01	0.704	0.18	C*12:02–B*27:04	0.141	0.72
C*01:02–B*15:01	0.626	−0.53	C*15:02–B*15:01	0.141	−0.40
C*03:03–B*15:07	0.610	0.93	C*08:03–B*15:01	0.136	0.03
C*03:02–B*58:01	0.568	1.00	C*03:03–B*55:04	0.120	0.91
C*04:01–B*56:01	0.537	0.61	C*07:02–B*40:02	0.115	−0.89
C*07:02–B*15:01	0.500	−0.48	C*04:01–B*15:27	0.109	1.00
C*06:02–B*37:01	0.495	1.00	C*04:01–B*48:01	0.109	−0.14

HF, haplotype frequencies; HLA, human leucocyte antigen; RD, relative linkage disequilibrium value.

a C‐B haplotypes with HF >0.1% are listed.

#### Observed recombination

A total observation number of recombination events were 136 in 134 families. These were divided into two groups: (i) the haplotypes of 103 parents (75.7%) could be determined, (ii) the haplotypes of 33 parents (24.3%) could not be determined and thus were inferred. Table 6 summarizes the observation number of recombination events in informative families which contain the parents and three or more children. The genotypes of these parents are heterozygote at all loci of HLA‐A, ‐B, ‐C, and ‐DRB1. Group (ii) is not included in these informative families. The transmission of recombination (%R/T) shows the HLA‐A‐DRB1 recombination probability as 1.08% per child. Furthermore, Table 6 also indicates the recombination probabilities of HLA‐A‐C and B‐DRB1 are 0.54%.

**Table 6:** Number of HLA–A/C, C/B, B/DRB1 recombination evens by each family structure

	Number of children	Number of families	A/C^a^ *n* (%R/T)	B/DRB1^a^ *n* (%R/T)	A/DRB1^a^ *n* (%R/T)
Parents	5	5	b	b	b
	4	28	2 (0.89)	1 (0.45)	3 (1.34)
	3	233	7 (0.50)	8 (0.57)	15 (1.07)
Total	836	266	9 (0.54)	9 (0.54)	18 (1.08)

HLA, human leucocyte antigen.

a X/Y, number of recombination between X and Y; %R/T, % of recombination per transmission.

b No observation of recombination.

#### Comparison to the result by MLE

Table 7 shows the haplotype frequencies of HLA‐A‐C‐B‐DRB1 based on family study (result‐FS) and based on MLE (result‐MLE) with frequencies more than 0.5%. In the frequent haplotypes with frequencies not less than 0.12%, result‐MLE tends to be higher than result‐FS. In the low‐frequent haplotypes with less than 0.12%, result‐MLE tends to be lower than result‐FS. In addition, result‐MLE could not be detected in 585 haplotypes of the 2099 haplotypes with frequencies less than 0.12% in result‐FS.

**Table 7:** Comparison to maximum likelihood estimation (n = 4500)^a^

A–C–B–DRB1	FS (%)	MLE (%)
A*24:02‐C*12:02‐B*52:01‐DRB1*15:02	8.144%	8.298%
A*33:03‐C*14:03‐B*44:03‐DRB1*13:02	4.444%	4.478%
A*24:02‐C*07:02‐B*07:02‐DRB1*01:01	3.689%	3.857%
A*24:02‐C*01:02‐B*54:01‐DRB1*04:05	2.344%	2.490%
A*02:07‐C*01:02‐B*46:01‐DRB1*08:03	2.044%	2.053%
A*11:01‐C*04:01‐B*15:01‐DRB1*04:06	1.322%	1.326%
A*24:02‐C*01:02‐B*59:01‐DRB1*04:05	1.100%	1.167%
A*11:01‐C*01:02‐B*54:01‐DRB1*04:05	0.833%	0.815%
A*26:01‐C*03:04‐B*40:02‐DRB1*09:01	0.778%	0.892%
A*24:02‐C*08:01‐B*40:06‐DRB1*09:01	0.733%	0.830%
A*24:02‐C*14:02‐B*51:01‐DRB1*09:01	0.722%	0.803%
A*31:01‐C*14:02‐B*51:01‐DRB1*08:02	0.589%	0.619%
A*33:03‐C*14:03‐B*44:03‐DRB1*08:03	0.578%	0.589%
A*26:02‐C*08:01‐B*40:06‐DRB1*09:01	0.556%	0.526%
A*24:02‐C*01:02‐B*46:01‐DRB1*08:03	0.522%	0.504%
A*02:01‐C*03:04‐B*13:01‐DRB1*12:02	0.511%	0.502%

FS, haplotype frequencies based on family study; MLE, haplotype frequencies estimated by maximum likelihood estimation.

a Four‐locus haplotypes with frequencies >0.5% in result‐FS are listed.

On the allele data used for this comparison, four loci showed Hardy–Weinberg Equilibrium: the *P*‐values of the exact test at HLA‐A, ‐B, ‐C, and DRB1 loci were 0.3184, 0.2557, 0.1449, and 0.4998, respectively.

## Discussion

The AF presented in Table 2 show the high frequency of A*24:02 at 36.48%; therefore, around 60% of the population could be administered peptide vaccines such as WT1 peptide vaccine presented by HLA molecules coded A*24:02 23, 24. Moreover, around 80% of the population could be administered the vaccines 25, 26 presented by HLA molecules coded by one of three alleles as A*24:02 (36.5%), A*02:01 (11.6%), and A*02:06 (9.1%).

The haplotype frequencies are also characteristic of Japanese population. The haplotypes of high frequencies are well conserved. Approximately one third (38%) of Japanese population carry the five most common haplotypes. We believe that this is strongly influenced by the founder effect: the ancestors having the common haplotypes migrated to Japan, thus, their haplotypes have been concentrated. While searching in the AFND 22, we found that the common haplotypes are homologous to those residing in the neighboring countries, especially in South Korea 11, 12, 27, 28, 29, 30, 31. In Korean population, the frequent haplotypes are similar to the ones in Japanese population. The three major Japanese haplotypes A*24:02‐C*12:02‐B*52:01‐DRB1*15:02, A*33:03‐C*14:03‐B*44:03‐DRB1*13:02, and A*24:02‐C*07:02‐B*07:02‐DRB1*01:01 have also high frequencies in Korea (1.9%, 4.2%, 2.9%, respectively) 27, suggesting the migration of some ancestors though the Korean Peninsula.

The haplotype analysis not only helps in understanding the history of human migration but also in matching for unrelated donor searches for HSCT. In JMDP, the HLA compatibility with a donor is evaluated by both serotype and genotype. Furthermore, allele matching is a better evaluation of compatibility compared to the serotype matching; the rejection and GVHD risk of bone marrow transplant (BMT) have been found to be lower with allele‐level matching compared to the serotype‐level matching 3, 32. Accordingly, focusing on the alleles in every serotype, A2, A26, etc. increases the possibility of allele mismatch in spite of serotype match (Table 2). As shown in Table 4, HLA‐A2, B61, B15, and DR4 especially increase the allele mismatch risk. However, compared with South Korean haplotype analysis, South Korea has 15 sets of haplotypes to pay attention to in HSCT matching 27 but Japan has eight sets (Table 4). It also shows that for most HLA haplotypes at the serotype‐level, HLA haplotype matching is almost allele‐level matching in HSCT between the Japanese.

Although HLA‐C locus is not indispensable for registering information in HSCT, HLA‐C allele matching is important 32, 33. Even without information of donor HLA‐C allele, the C‐B haplotypes can predict HLA‐C allele, although not always, because of well‐conserved C‐B linkage (Table 5); the conservation is possible due to short genetic linkage distance. In other words, HLA‐B allele matching increases the possibility of HLA‐C allele matching. Accordingly, analysis in the HLA distribution in Japanese population may contribute in planning the strategies of HLA matching for HSCT.

Result‐MLE was similar to result‐FS (Table 7). Table 7 indicates the haplotype frequencies estimated by the software are very similar to real family‐derived haplotypes. If the detection of the low frequency haplotypes is needed, determination of haplotypes by descent or a large sample size appears to be necessary.

In conclusion, this study of determination of HLA allele and haplotype frequencies using family samples not only serves as a tool for elucidating linkage of each HLA locus but also acts as a tool in detecting HLA gene mutations in human germ cells such as recombination. The data obtained in this study will be useful in various fields such as anthropology, transplantation therapy, and disease association studies.

## Conflicts of interest

The authors have no conflicts of interest.
